# Deprivation of Dietary Fiber Enhances Susceptibility of Piglets to Lung Immune Stress

**DOI:** 10.3389/fnut.2022.827509

**Published:** 2022-02-10

**Authors:** Yi Yang, Xuemei Jiang, Xuelin Cai, Lijia Zhang, Wentao Li, Lianqiang Che, Zhengfeng Fang, Bin Feng, Yan Lin, Shengyu Xu, Jian Li, Xilun Zhao, De Wu, Yong Zhuo

**Affiliations:** State Key Laboratory for Animal Disease-Resistant Nutrition of the Ministry of Education of China, Animal Nutrition Institute, Sichuan Agricultural University, Chengdu, China

**Keywords:** dietary fiber, lung immune stress, intestinal microbes, short-chain fatty acid, piglet

## Abstract

Growing evidence suggests that dietary fiber enhances short-chain fatty acid (SCFA) producing gut microbes, improving lung immunity against invading pathogens *via* the gut–lung axis. This study investigated the effects of dietary fiber on lung immune stress after challenge with complete Freund's adjuvant (CFA) containing killed *Mycobacterium tuberculosis*. Thirty-six healthy hybrid Duroc, Landrace, and Yorkshire male piglets (9.7 ± 1.07 kg, 35 ± 3 days) were randomly fed a low fiber (LF) diet formulated with semipurified corn starch, soy protein concentrate, and fishmeal or a high fiber (HF) diet composed of 1,000 g LF diet plus 20 g inulin, and 100 g cellulose. Piglets were housed individually in the metabolism cages with eighteen replicates per group, with one pig per cage. All the piglets received similar levels of digestible energy and lysine and had similar weight gain. After dietary treatment for 28 days, nine piglets per group were intravenously administered CFA (0.4 mg/kg) or an equivalent amount of sterile saline in a 2 × 2 factorial arrangement. In piglets fed the LF diet, CFA caused lung damage and elevated serum C-reactive protein and relative mRNA expression of genes related to lung inflammation (*NLRP3, Casp1, ASC, IL1*β*, IL18, Bax*). Compared with the LF diet, the HF diet increased bacterial diversity and *Deferribacteres* (*p* = 0.01) in the phylum level and *unidentified_Ruminococcaceae* (*p* = 0.03) and *Catenisphaera* (*p* < 0.01) in the genus level. The HF diet improved increased short-chain fatty acids in feces, blood, cecal, and colonic digesta; reduced lung damage; and promoted lung recovery. Overall, dietary fiber deprivation enhanced the risk of piglets to lung immune stress, demonstrating the importance of dietary fiber in gut–lung health.

## Introduction

Asthma is a chronic inflammatory disease of the airway, and more than 300 million people worldwide are threatened by this disease (1). Asthma is also the most prevalent childhood disease in Western countries (2, 3). In the global pig industry, respiratory illnesses, including porcine respiratory diseases, account for up to 15% of mortality in pigs (4). Changes in nutritional status may be essential to prevent respiratory diseases (5). Numerous studies suggest that the intestinal microbiota is a link between respiratory diseases and the immune system, and many recent studies have identified the intestinal microbiota as a potential therapeutic target for the prevention of asthma and atopic diseases (6–9). Traditional Chinese medicine (TCM) theory suggests that the lung and the large intestine are connected *via* both physical contact and pathological interactions (10, 11). The earliest record of this theory is found in the Huangdi Neijing (the Yellow Emperor's Canon of Internal Medicine), which was first published over 2,000 years ago (12). Today, the treatment of intestinal diseases is known to prevent lung diseases, such as asthma, adult respiratory distress syndrome, and chronic obstructive pulmonary disease (COPD) (13–15). In addition, improvements in lung health are closely related to the treatment of intestinal disorders, such as colitis, irritable bowel syndrome, inflammatory bowel disease, and others (16, 17). Together, these studies demonstrate that gut–lung communication plays a role in the control of overall health.

A previous study found that high dietary fiber intake was inversely associated with the risk of COPD and it included smokers (18). Dietary fiber also plays a beneficial role in the prevention of gastrointestinal inflammatory diseases and colon cancer (19). However, little is known about the effects of dietary fiber intake on parenteral inflammation. Dietary fiber is a complex carbohydrate composed of soluble and insoluble components that are resistant to endogenous digestive enzymes and, therefore, require microbial metabolism to produce short-chain fatty acids (SCFAs) (20). Insoluble fibers (e.g., cellulose) have important leavening properties, whereas soluble forms (e.g., inulin) are fermented by certain types of intestinal bacteria to produce biologically active byproducts (21). SCFAs are among the most abundant metabolites of fiber and may serve as a fuel source for intestinal epithelial cells or play a key role in regulating intestinal morphology and function (22). In addition, SCFAs are, in turn, an energy source for certain bacterial species and can alter the composition of the microbiome (23, 24). As a major metabolite of dietary fiber, SCFAs shape the immune environment and influence the severity of allergic inflammation in asthmatic mice, which is thought to be caused by binding to the endogenous receptor GPR41 (25). Recent findings showed that fiber or its metabolite acetate led to a marked suppression of allergic asthma in both mothers and their offsprings by enhancing regulatory T (Treg) cell numbers and function (26). Schuijt confirmed that the diversity of the intestinal microbiota was a key factor affecting the ability of alveolar macrophages to phagocytose *Streptococcus pneumonia* (27). These studies indicate that there is a close relationship between intestinal health and the immune response in the lung, but it is unclear whether dietary fiber can alleviate immune stress in piglets *via* the intestinal microbiota.

We hypothesized that dietary fiber may attenuate the lung immune stress by regulating the intestinal microbiota. Pigs are widely used as a model of human pathology owing to similarities in their metabolic physiology, gastrointestinal anatomy and physiology, and nutrient digestibility (28). Therefore, pigs were used as the experimental model in this study.

## Materials and Methods

### Experimental Design and Animal Management

The research protocol was approved by the Animal Care and Use Committee of the Animal Nutrition Institute of Sichuan Agricultural University (No. SICAU-2015-034) and was conducted in accordance with the Guidelines for the Care and Use of Laboratory Animals of the National Research Council of the United States.

A total of 36 crossbred Duroc, Landrace, and Yorkshire male piglets (35 ± 3 days of age) with an initial average body weight (BW) of 9.7 = ± 1.07 kg were used. Piglets were randomly allocated to two dietary treatments with 18 replicate pens per group. Piglets were housed individually in metabolism cages and fed either a low dietary fiber (LF) diet formulated with semipurified corn starch, soy protein concentrate, and fishmeal or a high dietary fiber (HF) diet formulated by replacing 12% of the LF diet with inulin (ZTH Tech, Beijing, China) and cellulose (Guangxi Shangda Tech Co., Nanning, China) (1:5 ratio) (Table 1). The nutrient content of the diet was formulated to meet or exceed the nutrient requirements of swine recommended by the National Research Council (2012) (29). During the first week of the experiment, the room temperature was maintained at 26–28°C, then was reduced to 22–24°C during the last week of the experiment. Humidity was maintained between 50 and 60% throughout the experiment. The experiment period was 32 days, and each piglet was provided with food and water *ad libitum*.

**Table 1:** Ingredients composition and nutrient level of the diet (as-fed basis).

**Item**	**Composition, %**	
	**LF**	**HF**
**Ingredients**		
Casein (88.0% CP)	3.6	3.6
Isolated soy protein (84.8% CP)	4	4
Skimmed milk powder (34.8% CP)	10	10
Whey powder	10	10
Corn starch	60.97	60.97
Fishmeal (65.0% CP)	8.5	8.5
Soybean oil	1	1
Limestone	0.3	0.3
L-Lysine HCl (98.5%)	0.29	0.29
DL-Methionine (98.5%)	0.24	0.24
L-Threonine (98.5%)	0.36	0.36
L-Tryptophan (98.5%)	0.02	0.02
Valine (99.0%)	0.11	0.11
Choline chloride (50%)	0.2	0.2
NaCl, feed-grade	0.3	0.3
Minerals premix^a^	0.1	0.1
Vitamin premix^b^	0.01	0.01
Inulin (90.0%, total dietary fiber)	–	2
Cellulose (91.5%, total dietary fiber)	–	10
Total^c^	100	112
**Nutrient composition**		
DE, Mcal/kg	3.86	3.45
CP, %	17.56	15.68
Calcium, %	0.71	0.64
Available phosphorus, %	0.45	0.40
SID Lysine, %	1.4	1.25
SID Methionine + Cysteine, %	0.79	0.71
SID Threonine, %	0.87	0.78
SID Tryptophan, %	0.23	0.21
SID Valine, %	0.91	0.81
Total dietary fiber, %	0	10.95

*DE, digestible energy; SID, standardized ileal digestibility; fiber diet Add 2 kg of inulin and 10 kg of cellulose to each 100 kg of basal diet*.

a *Mineral premix provided per kilogram of diet: Cu 6 mg as CuSO_4_·5H_2_O; Fe 100 mg as FeSO_4_·7H_2_O; I 0.14 mg as KI; Zn 100 mg as ZnSO_4_·7H_2_O; Mn 20 mg as MnSO_4_·H_2_O; Se 0.3 mg as Na_2_SeO_3_·5H_2_O*.

b *Vitamin premixes provided per kilogram of diet: vitamin A, 3,000 IU; vitamin D_3_, 1,000 IU; vitamin E, 8 IU; vitamin K_3_, 1 mg; vitamin B_1_, 1 mg; vitamin B_2_, 2.5 mg; vitamin B_6_, 1.2 mg; vitamin B_12_, 0.12 mg; nicotinic acid, 10 mg; pantothenic acid, 5 mg; folic acid, 0.5 mg; biotin, 0.5 mg*.

c *The HF diet was formulated by adding 2 kg of inulin and 10 kg of cellulose to each 100 kg of LF diet*.

### Lung Stress Challenge Tests

After dietary treatment for 28 days, half of the piglets in each group were intravenously infused in the ear vein with complete Freund's adjuvant (CFA) containing killed *Mycobacterium tuberculosis* (0.4 mg/kg BW, F-5881; Sigma-Aldrich) or an equivalent amount of sterile saline. Respiratory rate was recorded at 1 and 24 h after CFA challenge, with per exhalation and inhalation as the number of one breath.

### Sample Collection and Measurements

The BW and feed intake of each piglet were measured weekly. The average daily feed intake (ADFI) and nutrient intake were calculated. On day 32 of the experiment (4 days post-CFA administration), the piglets were euthanized and slaughtered by intravenous injection of 50 mg of sodium pentobarbital per 1 kg of body weight.

Fasting blood samples were collected *via* venipuncture from each piglet before the morning meal on days 28 and 30 of the experiment at 8:00 am. Blood samples were centrifuged at 3,000 × *g* at 4°C for 30 min to collect serum, which was stored at −20°C for further analysis. Fresh feces were collected by massaging the rectum of each piglet on days 7, 14, 21, and 28 of the experiment. Freshly collected samples were transported in dry ice and stored at −80°C until analysis.

Samples of the duodenum, jejunum, ileum, and colon, ~2 cm in length, and the right middle lobe of the lung were collected and stored in 4% paraformaldehyde solution for histological analysis. A subset of these tissues was snap-frozen in liquid nitrogen and stored at −80°C until further analysis. Finally, samples of digesta (2 mL) from the midsections of the colon and caecum were collected immediately after their removal, frozen in liquid nitrogen, and stored at −80°C.

### Histological Analysis

**Figure 1:**
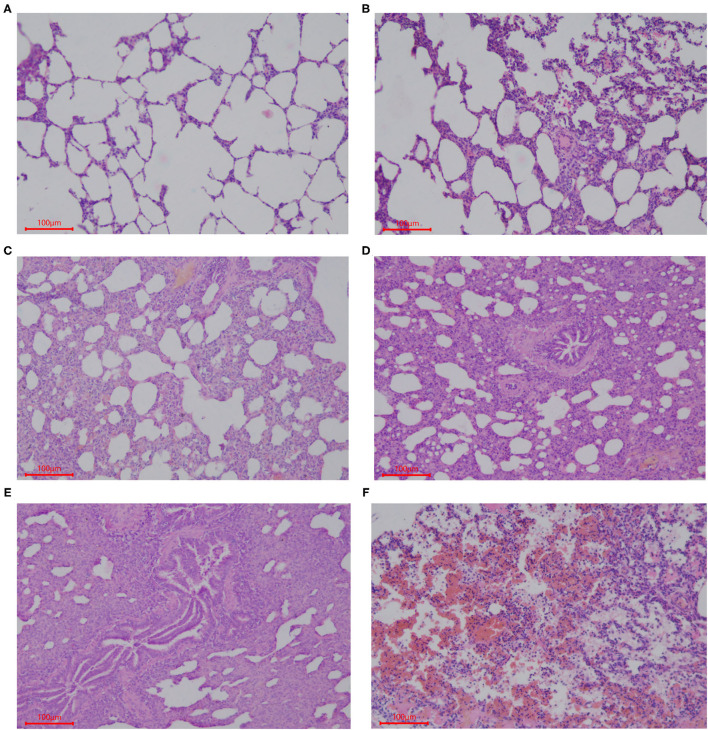
Scoring criteria of lung pathological damage after CFA challenge. The lung injury of piglets in each group was graded by the following description: **(A)** 1, normal lung tissue structure, thin and non-proliferating alveolar wall, focal hyperplasia, unconnected hyperplastic nodules, and no exudate or red blood cells in the alveolar cavity; **(B)** 2, with pulmonary interstitial hyperplasia, presence of two ocal nodular hyperplasia, the nodules connected by proliferated alveolar septal, with local alveolar wall Telangiectasia congestion or congestion, and the presence of red blood cells in alveolar cavity; **(C)** 3, mild pulmonary interstitial hyperplasia, diffuse and widening alveolar septum with a small amount of inflammatory cell infiltration, narrow alveolar space; **(D)** 4, moderate hyperplasia of the interstitium, reduced number of alveoli, severe stenosis in the alveolar cavity accompanied by a large number of inflammatory cells; **(E)** 5, severe pulmonary interstitial hyperplasia and fusion, large areas of alveolar stenosis or disappearance accompanied by a large number of inflammatory cells; **(F)** 6, destruction of lung tissue, severe pulmonary hemorrhage, large numbers of red blood cells and serous exudate in the alveolar cavity and diffuse infiltration of a large number of inflammatory cells.

Samples of the intestinal and lung tissues were taken from the fixation solution and dehydrated using increasing concentrations of ethanol and chloroform, followed by treatment with paraffin wax. Sections (5 μm) of each tissue were cut and mounted on glass slides, which were stained using hematoxylin and eosin (HE). The histological structures of the tissue were analyzed and photographed using a digital camera (Nikon Eclipse 50). Five slides of each sample were prepared with three 5-μm thick sections placed on each slide, and the length of 20 well-oriented villi and crypts from each section was assessed (Image Pro Plus 6.0). Villi height was measured from the tip of the villi to the point between each villus, the crypt depth was measured from the valley between each villus to the basement membrane, and then the villus-crypt ratio (VCR) was calculated (30). Lung tissue sections were stained with HE, and histopathological changes were observed under a microscope (Nikon Eclipse 50). The entire histogram was completely observed. Normal tissues and obvious lesions were photographed using a microscope imaging system, and the scoring system was developed based on the histology of tissues in this experiment. Grading criteria were based on the degree of lung injury in piglets, and lung pathological damage was scored according to Figure 1.

### SCFA Analysis

SCFA concentrations in fecal samples and intestinal chyme were determined using gas chromatography (manual injection, flame ionization detector, 10 μL microinjector; Varian CP-3800). Briefly, ~0.7 g of each fecal and chyme sample were diluted with 1.5 mL ultrapure water, mixed, and placed at 4°C for 30 min. Afterward, all samples were centrifuged at 15,000 × *g* for 15 min at 4°C to obtain 1 mL of supernatant, to which 0.2 mL of 25% metaphosphoric acid and 23.3 μL crotonic acid (210 mmol/L, internal standard) were added. The solution was mixed by vortexing, placed at 4°C for 30 min, and centrifuged at 15,000 × *g* for 10 min at 4°C. A total of 0.3 mL supernatant was obtained and mixed with 0.9 mL methanol, and then filtered through a 0.22-μm filter (Millipore Co.).

The concentrations of SCFA in the serum were analyzed using gas chromatography. A total of 400 μL serum was mixed with 50 μL metaphosphoric acid (25% w/v) and 4 μL crotonic acid (21 mmol/L, internal standard). After mixing, the solutions were incubated at 4°C for 30 min, followed by centrifugation at 15,000 × *g* for 10 min at 4°C. A total of 100 μL supernatant was obtained and mixed with 100 μL methanol and then centrifuged at 15,000 × *g* for 15 min. The supernatant was collected and filtered through a 0.22-μm filter (Millipore Co.).

### RNA Extraction and Gene Expression Analysis

Total RNA was extracted from frozen lung tissue using TRIzol reagent (Invitrogen, Takara Bio Inc.) according to the manufacturer's instructions. The quality and purity of RNA samples were assessed by electrophoresis on 1.0% agarose gels and by using a nucleic acid analyzer (A260/A280, DU-800; Beckman Coulter, Inc.), respectively. Subsequently, RNA was incubated at 37°C for 15 min, followed by reverse transcriptase inactivation at 85°C for 5 s using a Prime Script RT^TM^ reagent kit (RR047A; Takara Bio Inc.). Quantitative RT-PCR was performed using an ABI-7900HT instrument (Applied Biosystems). Oligonucleotide primers were used to detect the expression of the target genes and the reference gene (β-actin) using the SYBR green system (RR820A; Takara Bio Inc.). The sequences of primers used and the lengths of the products are presented in Table 2. The reaction mixture (10 μL) contained 5 μL fresh SYBR® Premix Ex TaqII (Tli RNase H Plus), 0.2 μL ROX Reference Dye II (50×), 0.8 μL primers, 1 μL RT products, and 3 μL diethylpyrocarbonate-treated water. The following PCR protocol was used: one cycle at 95°C for 30 s; 40 cycles at 95°C for 5 s, and 60°C for 31 s; and one cycle at 95°C for 15 s, 60°C for 1 min, and 95°C for 15 s. The standard curve of each gene was run in duplicate and three times to obtain reliable amplification efficiency values. The correlation coefficients of all standard curves were >0.99, and the amplification efficiency values were between 90 and 110%. At the end of each amplification, a melting curve analysis was performed to determine amplification specificity. The β-actin transcript was used to standardize the results by eliminating variations in mRNA and complementary DNA quantity and quality, and the levels of each mRNA transcript were expressed as ratios to β-actin mRNA. Relative quantification of gene expression among the treatment groups was analyzed using the 2^−ΔΔCt^ method (31). The relative mRNA expression levels of each target gene were normalized to the LF group.

**Table 2:** Primer sequences of the target and reference genes^a^^,^^b^.

**Genes symbol**	**Nucleotide sequence of primers (5'−3')**	**Product size**	**Accession**
*β-actin*	F: GGCGCCCAGCACGAT	66	XM_021086047.1
	R: CCGATCCACACGGAGTACTTG		
*GPR41*	F: TCTTCACCACCGTCTATCTCAC	398	NM_001315601.1
	R: CACAAGTCCTGCCACCCTC		
*GPR43*	F: CTGCCTGGGATCGTCTGTG	249	XM_021093196.1
	R: CATACCCTCGGCCTTCTGG		
*Caspase3*	F: TCTAAGCCATGGTGAAGAAGGAAAAA	112	NM_214131.1
	R: GGGTTTGCCAGTTAGAGTTCTACAG		
*IL-10*	F: CACGGCCTTGCTCTTGTTTT	148	NM_214041.1
	R: CCTGGAAGACGTAATGCCGA		
*TGF-β*	F: CTGACCCGCAGAGAGGCTAT	102	NM_214015.2
	R: AGAATTGAACCCGTTAATTTCCACG		
*Bax*	F: TGACGGCAACTTCAACTGGG	143	XM_013998624.2
	R: GCAGCCGATCTCGAAGGAAGT		
*Bcl2*	F: GAGGATTGTGGCCTTCTTTGAGT	155	XM_021099593.1
	R: CATCCCAGCCTCCGTTATCC		
*Foxp3*	F: TCAGACCAACAGGGAGCCAA	88	XM_021079539.1
	R: TCAAGGAGGAAGAGGAGGCG		
*HDAC9*	F: CAACAGAACGGATGGGGTGG	128	XM_021102511.1
	R: GGTCTAAAGGCGAGATGGGC		
*NLRP3*	F: GGAGGAGGAGGAAGAGGAGATA	147	NM_001256770.2
	R: AGGACTGAGAAGATGCCACTAC		
*IL18*	F: AGTAACCATCTCTGTGCAGTGT	155	NM_213997.1
	R: TCTTATCATCATGTCCAGGAAC		
*Caspase1*	F: GAAGGAGAAGAGGAGGCTGTT	268	NM_214162.1
	R: AGATTGTGAACCTGTGGAGAGT		
*ASC*	F: ACAACAAACCAGCACTGCAC	126	XM_003124468.5
	R: CTGCCTGGTACTGCTCTTCC		
*IL1β*	F: TCTGCCCTGTACCCCAACTG	64	XM_021085847.1
	R: CCAGGAAGACGGGCTTTTG		

a *F, forward primer sequence (5′ → 3′); R, reverse primer sequence (5′ → 3′)*.

b *Gene: GPR41, G protein-coupled receptor 41; GPR43, G protein-coupled receptor 43; Caspase3, Cysteine-requiring aspartate protease 3; IL 10, Interleukin 10; TGF-β, transforming growth factor-β; Bax, BCL2-Associated X; Bcl2, B cell lymphoma 2; Foxp3, Forkhead box P3; HDAC9, Histone deacetylase 9; NLRP3, Nod-like receptor, pyrin domain 3 containing; IL 18, Interleukin 18; Caspase1, Cysteine-requiring aspartate protease 1; ASC, Apoptosis-associated speck-like protein containing CARD; IL-1β, Interleukin 1β*.

### Microbial Analysis

Total genomic DNA was extracted from the colonic digesta using an Omega DNA stool kit (Omega Bio-Tek) according to the manufacturer's instructions. Before sequencing, the concentration and purity of the extracted genomic DNA were measured. The integrity of the extracted genomic DNA was determined by electrophoresis on a 1% (w/v) agarose gel. Primer design and bioinformatics analyses were performed by Novogene using the Illumina HiSeq platform with paired-end sequencing. Raw data were screened and assembled using FLASH (V1.2.7) and QIIME (V1.7.0) software packages. Chimera sequences were removed using the UCHIME algorithm to create an “effective sequence” collection for each sample. UPARSE software (V7.0.1001) was used for sequence analysis and operational taxonomic unit (OTU) determination with an identity threshold of 97%. We selected a representative sequence for each OTU and used RDP Classifier (V2.2) to assign taxonomic data to each representative sequence. Taxonomy classifications were assigned using RDP Classifier and the Greengenes database. Taxon abundance patterns for each sample were determined at the phylum, class, order, family, and genus levels. All analyses from clustering to alpha diversity analyses [observed_species, Chao1, abundance-based coverage estimator (ACE), Simpson and Shannon indices] and beta diversity of principal component analysis (PCA) were calculated using QIIME software (V1. 7. 0) (32). The correlations between lung pathology score and microbial abundance at the phylum and genus level were evaluated by Pearson correlation analysis and visualized diagrams were created using R (V2. 15. 3). Alpha and beta community diversity were calculated with QIIME (V1. 7. 0). R (V2. 15. 3) and GraphPad Prism (V8. 0. 2) were used to create visualized diagrams.

### Western Blot Analysis of Protein Expression

Protein expression measurements were performed as previously described (33). Briefly, frozen lung tissues were ground in liquid nitrogen, homogenized in cell lysis buffer (Beyotime Biotechnology) supplemented with protease inhibitor cocktail (Roche), and centrifuged at 12,000 × g for 30 min at 4°C. The supernatant was collected for protein concentration measurements using a BCA protein assay kit (Thermo Scientific) and a plate reader. Equal amounts of protein lysate (40 μg) were separated on a 10% sodium dodecyl sulfate-polyacrylamide gel electrophoresis (SDS-PAGE) gel after boiling at 95°C for 10 min and then transferred to a polyvinylidene fluoride membrane (Bio-Rad Laboratories). The membrane was blocked in TBST buffer (50 mM TRIS-HCl, 150 mM NaCl, 0.1% Tween, pH 7.6) supplemented with 5% bovine serum albumin (Sigma-Aldrich) at room temperature for 1.5 h, followed by overnight incubation at 4°C with diluted primary antibodies against BAX (1:1,000; Abcam), BCL-2 (1:500; Abcam), caspase-3 (1:1,000; Cell Signaling Technology), NF-κB (1:1,000; Cell Signaling Technology), and β-actin (1:1,000; TRANS). After 1 h of incubation with horseradish peroxidase-linked anti-mouse IgG secondary antibody (1:2,000; Cell Signaling Technology) and anti-rabbit IgG (1:2,000; Cell Signaling Technology) at room temperature, chemiluminescence detection was performed using the ECL Plus TM Western Blotting Detection System on a Molecular Imager ChemiDoc XRS+ System (Bio-Rad Laboratories) according to the manufacturer's instructions. The relative expression of the target protein was normalized using β-actin as the internal protein, and the normalized values were used to compare target protein expression across groups.

### Statistical Analysis

Data were analyzed using the MIXED procedure (SAS 9.4 Inst. Inc., Cary, NC) as a completely randomized design. The individual piglet served as the experimental unit for all the variables measured. The data before the CFA challenge were analyzed by the Student-*t*-test (SAS 9.4 Inst. Inc., Cary, NC). The statistical model for the tissue-specific CFA challenge was analyzed as a 2 × 2 factorial arrangement. The statistical model used included the main effects of piglet diet (LF or HF), challenge (CFA or sterile saline), and their associated 2-way interaction. The data were checked for normality using the PROC UNIVARIATE function of SAS. All data presented in the table are expressed as mean and ensemble standard error (SEM). The correlation analysis was performed by Pearson correlation tests. Values *p* < 0.01 was considered highly significant, *p* < 0.05 was considered a significant difference, and 0.05 ≤ *p* < 0.10 was considered a trend.

## Results

### Growth Performance

The effects of dietary fiber on the growth performance of the piglets are presented in Supplementary Table S1. Throughout the experiment, there were no significant differences in BW, ADFI or digestible energy, and lysine intake between piglets fed the HF and LF diets.

### SCFA Concentrations

Concentrations of acetate and propionate were higher in the feces and serum of piglets fed the HF diet compared with those fed the LF diet (Table 3, *p* < 0.01). Dietary fiber had no effect on fecal concentrations of butyrate or valerate, but it did increase fecal concentrations of valerate on days 21 and 28 (Table 3, *p* < 0.05). Dietary fiber tended to increase serum concentrations of valerate (*p* = 0.060). Regardless of CFA treatment, piglets fed the HF diet had significantly higher concentrations of acetate, propionate, and butyrate in their caecal digesta and colonic digesta compared to piglets fed the LF diet (Table 4, *p* < 0.01). Furthermore, serum concentrations of acetate and propionate were higher in piglets fed the HF diet than in piglets fed the LF diet (Table 4, *p* < 0.01).

**Table 3:** Effect of dietary fiber on the concentration of short-chain fatty acids in feces and serum of piglets^a^.

**Item**	**Diet**		**SEM**	***P*-value**
	**LF**	**HF**		
**Acetate**, **μmoL/g**				
7 d	29.27	34.45	2.591	0.169
14 d	28.61	42.27	2.109	<0.001
21 d	26.34	50.48	2.616	<0.001
28 d	32.25	54.46	2.238	<0.001
**Propionate**, **μmoL/g**				
7 d	5.40	10.40	0.789	<0.001
14 d	5.52	14.70	1.054	<0.001
21 d	6.38	14.17	1.166	<0.001
28 d	6.48	15.79	1.430	<0.001
**Butyrate**, **μmoL/g**				
7 d	8.54	6.63	1.034	0.193
14 d	7.23	7.73	0.847	0.684
21 d	7.11	9.73	1.123	0.112
28 d	6.49	8.55	0.980	0.157
**Valerate**, **μmoL/g**				
7 d	2.43	2.24	0.317	0.665
14 d	1.92	2.57	0.295	0.140
21 d	2.14	3.27	0.345	0.038
28 d	2.14	3.10	0.283	0.028
**Total SCFAs**, **μmoL/g**				
7 d	45.64	53.71	3.695	0.135
14 d	43.28	67.27	3.133	<0.001
21 d	41.96	77.75	3.865	<0.001
28 d	47.36	81.91	3.867	<0.001
**Serum SCFAs**, **μmoL/L**				
Acetate	47.69	53.13	1.12	0.00
Propionate	23.38	30.03	1.09	0.00
Butyrate	0.55	0.69	0.05	0.11
Valerate	3.94	4.71	0.27	0.06

a *Data are means ± SEM, n = 18 in each group*.

**Table 4:** Effects of dietary fiber on the content of short-chain fatty acids in chyme and serum after CFA challenge of piglets^c^.

**Item**	**LF**		**HF**		**SEM**	***P-*** **value**		
	**–CFA**	**+CFA**	**–CFA**	**+CFA**		**Fiber**	**CFA**	**Fiber × CFA**
**Cecal chyme**								
Acetate, μmoL/g	25.44^b^	25.06^b^	41.61^a^	43.47^a^	3.504	<0.001	0.834	0.751
Propionate, μmoL/g	7.48^ab^	3.95^b^	13.95^a^	12.53^a^	2.379	0.004	0.307	0.661
Butyrate, μmoL/g	3.29^b^	2.90^b^	5.92^a^	6.66^a^	0.633	<0.001	0.779	0.377
Valerate, μmoL/g	1.03	1.93	2.18	2.11	0.148	0.252	0.455	0.550
Total cecal chyme SCFAs, μmoL/g	34.93^b^	31.26^b^	57.08^a^	60.57^a^	3.965	<0.001	0.981	0.373
**Colonic chyme**								
Acetate, μmoL/g	27.92^b^	23.52^b^	41.88^a^	39.86^a^	2.220	<0.001	0.157	0.595
Propionate, μmoL/g	2.13^b^	2.79^b^	4.29^a^	3.30^a^	0.416	<0.001	0.426	0.442
Butyrate, μmoL/g	4.94^b^	3.92^b^	7.40^a^	8.55^a^	0.669	<0.001	0.935	0.148
Valerate, μmoL/g	1.37^b^	1.53^b^	1.85^ab^	2.48^a^	0.293	0.021	0.193	0.430
Total colonic chyme SCFAs, μmoL/g	35.81^b^	31.32^b^	52.48^a^	53.90^a^	2.822	<0.001	0.591	0.304
**Serum**								
Acetate, μmoL/L	52.39^b^	52.14^b^	57.91^ab^	61.72^a^	2.058	<0.001	0.393	0.331
Propionate, μmoL/L	27.03^a^	23.76^b^	29.73^a^	28.71^a^	1.116	0.002	0.063	0.323
Butyrate, μmoL/L	0.40	0.50	0.48	0.50	0.039	0.275	0.133	0.287
Valerate, μmoL/L	3.83	3.98	4.68	4.46	0.350	0.067	0.929	0.592
Total SCFAs, μmoL/L	83.64^b^	80.38^b^	92.82^a^	95.40^a^	2.764	<0.001	0.903	0.298

a, b *Mean values within a row with different superscript letters were significantly different (p < 0.05)*.

c *Data are means ± SEM, n = 9 in each group*.

### Small Intestine Morphology and Colonic Mucosal Thickness

**Figure 2:**
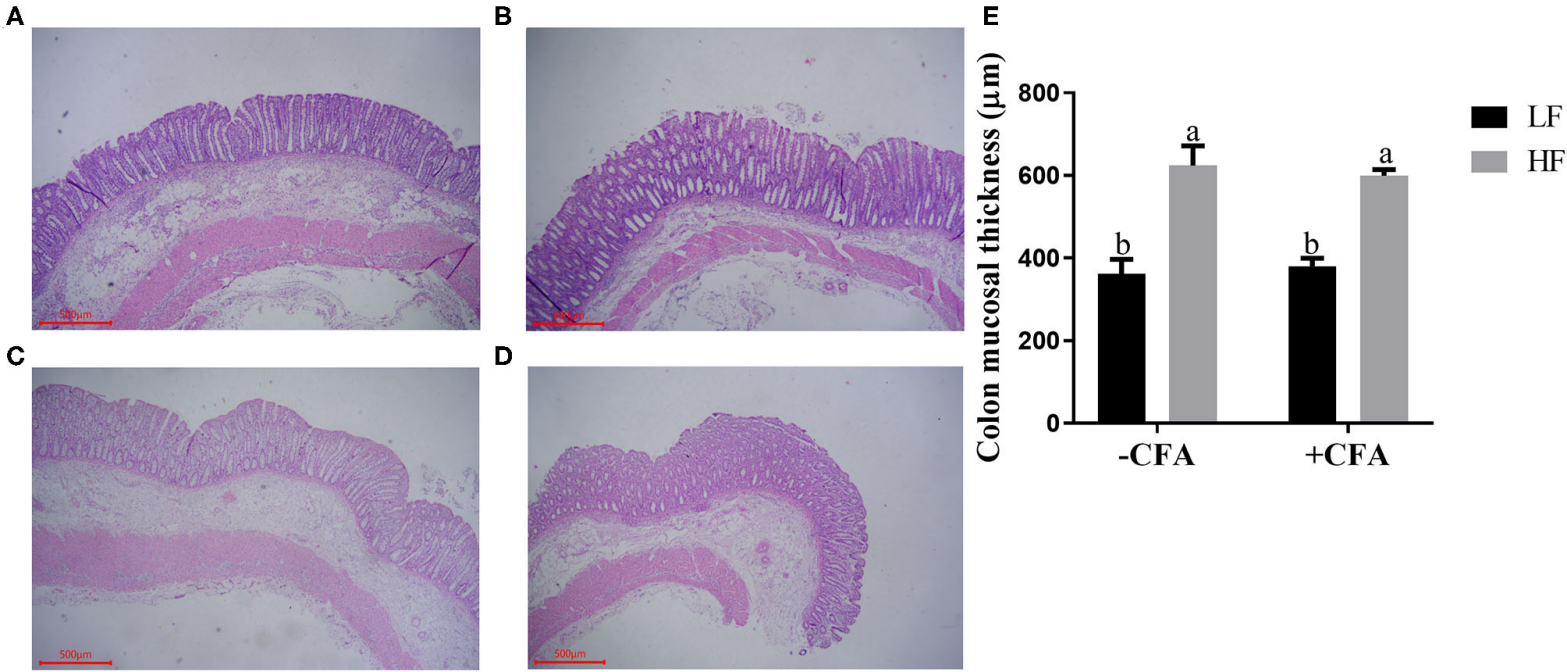
Effect of dietary fiber on colonic mucosal thickness of piglets. **(A)** LF without CFA challenge; **(B)** HF without CFA challenge; **(C)** LF with CFA challenge; **(D)** HF with CFA challenge. Original magnification 200×; stained with hematoxylin and eosin staining. ^a,b^Mean values within a row with different superscript letters were significantly different (*p* < 0.05). **(E)** Histogram of colonic mucosal thickness data.

No significant interaction effects between dietary fiber and CFA challenge were observed for villus height, crypt depth, or VCR in the duodenum and jejunum. However, the villus height and VCR in the ileum of piglets fed the HF diet increased (Supplementary Table S2, *P* < 0.05), and the colonic mucosa was thicker in piglets fed the HF diet than in those fed the LF diet (Figure 2, *p* < 0.01).

### Respiratory Rates, Serum C-Reactive Protein Concentrations and Lung Pathology of Piglets Challenged With CFA

**Figure 3:**
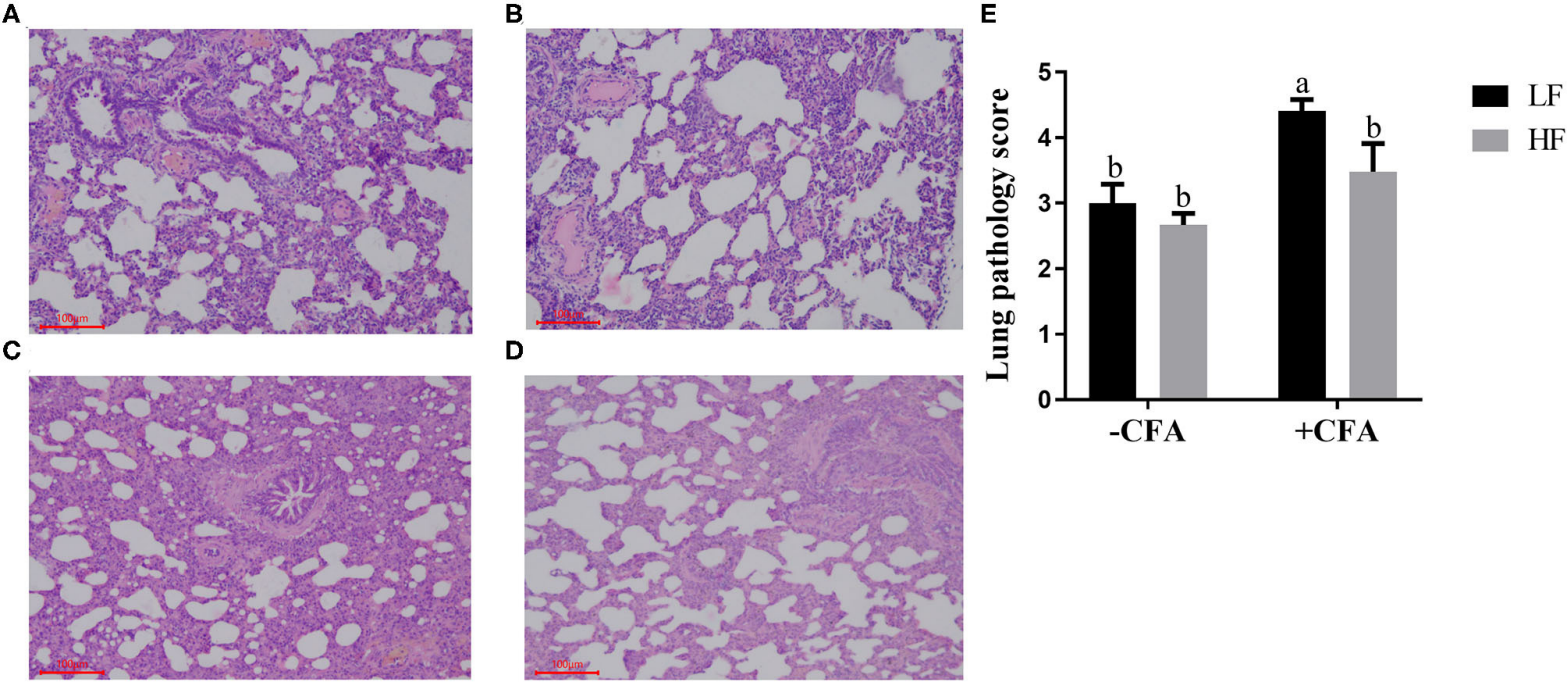
Effect of dietary fiber and CFA on pathological slices in lungs of piglets. **(A)** LF without CFA challenge; **(B)** HF without CFA challenge; **(C)** LF with CFA challenge; **(D)** HF with CFA challenge. Original magnification 200×; stained with hematoxylin and eosin staining. ^a,b^Mean values within a row with different superscript letters were significantly different (*p* < 0.05). **(E)** Histogram of lung pathology score data.

The CFA challenge significantly increased the respiratory rate (Table 5, *P* < 0.01) and the serum C-reactive protein concentrations (Table 5, *p* < 0.01). Compared with the LF diet treated with CFA, the HF diet with CFA treatment has a tendency to reduce the respiratory rate (Table 5, *P* = 0.065). Furthermore, CFA-induced lung pathological damage was lower in piglets fed the HF diet than in those fed the LF diet (Figure 3, *p* < 0.05).

**Table 5:** Effects of dietary fiber on respiratory rates and serum C-reactive protein in piglets after CFA challenge^c^.

**Item**	**LF**		**HF**		**SEM**	***P-*** **value**		
	**–CFA**	**+CFA**	**–CFA**	**+CFA**		**Fiber**	**CFA**	**Fiber × CFA**
1 h after the first treatment	53.11^b^	129.40^a^	58.22^b^	125.80^a^	4.251	0.860	<0.001	0.313
24 h after the first treatment	49.11^c^	87.40^a^	48.00^c^	76.40^b^	3.170	0.065	<0.001	0.128
1 h after the second treatment	55.78^c^	156.70^a^	57.56^c^	141.60^b^	5.246	0.213	<0.001	0.117
24 h after the second treatment	59.33^b^	75.40^a^	56.22^b^	72.00^a^	4.113	0.434	<0.001	0.972
Serum C-reactive protein	4.01^b^	11.26^a^	3.44^b^	11.3^a^	0.382	0.664	<0.001	0.723

a, b *Mean values within a row with different superscript letters were significantly different (p < 0.05)*.

c *Data are means ± SEM, n = 9 in each group*.

### Changes in Fecal Bacterial Diversity

**Figure 4:**
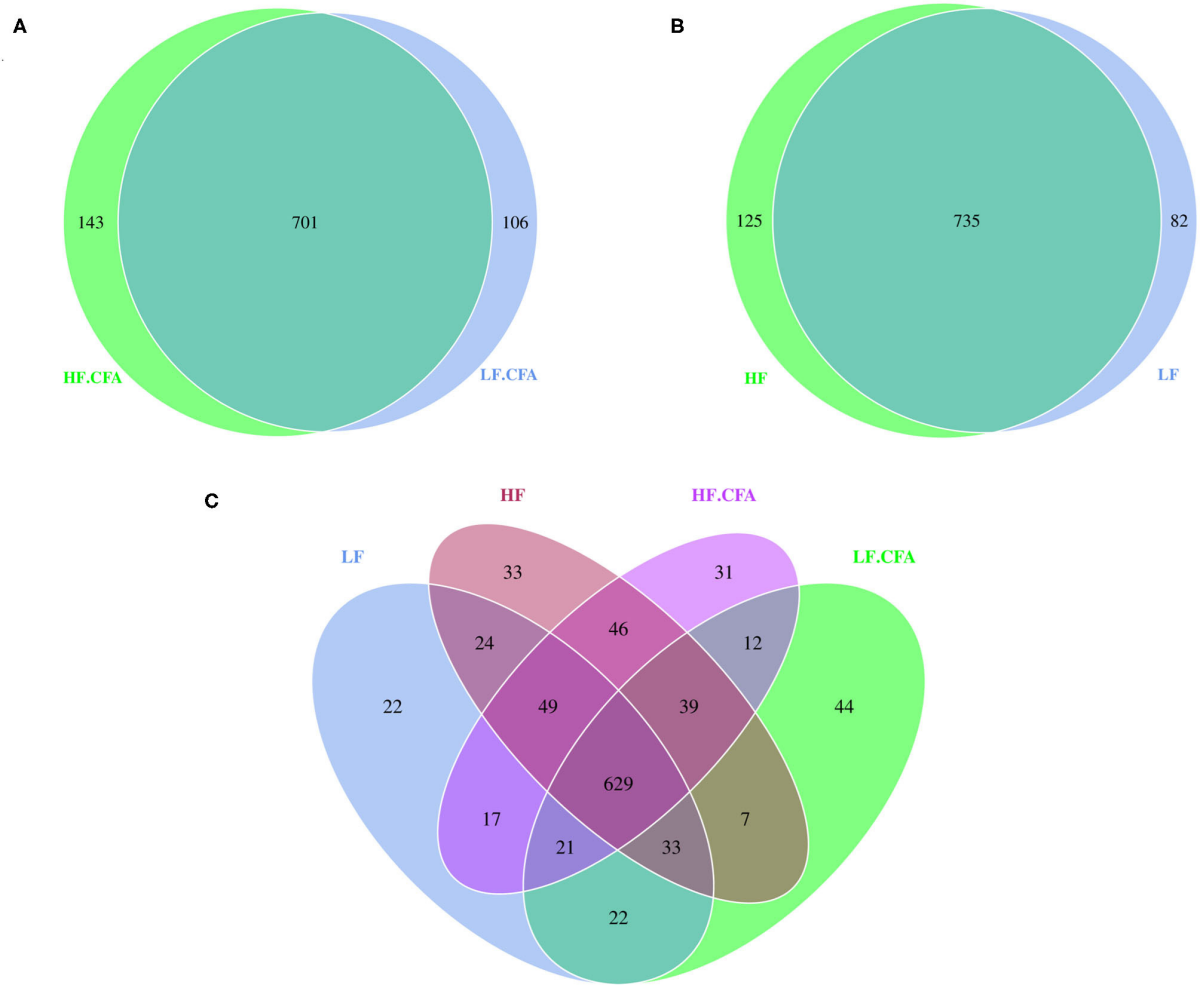
Microbiota comparison of the OTUs among treatments in the colon. The observed OTUs sharing ≥97% sequence similarity. **(A–C)** Venn diagrams describe the common and unique OTUs among four groups, respectively, HF, High fiber, no CFA; LF, Low fiber, no CFA; HF.CFA, High fiber with CFA challenge; LF.CFA, Low fiber with CFA challenge.

Based on the result of OUTs analysis obtained by clustering, Venn diagrams were used to evaluate the distribution of OTUs among the different groups. Venn diagrams (Figures 4A,B) showed that a total of 817, 860, 844, and 807 OTUs were observed in LF, HF, HF + CFA, and LF + CFA groups, respectively. The four groups shared the same 629 OTUs, but the OTUs were less in pigs on the LF diet than those on the HF diet (Figure 4C). There was no interaction effect on the numbers of observed species in each group.

**Figure 5:**
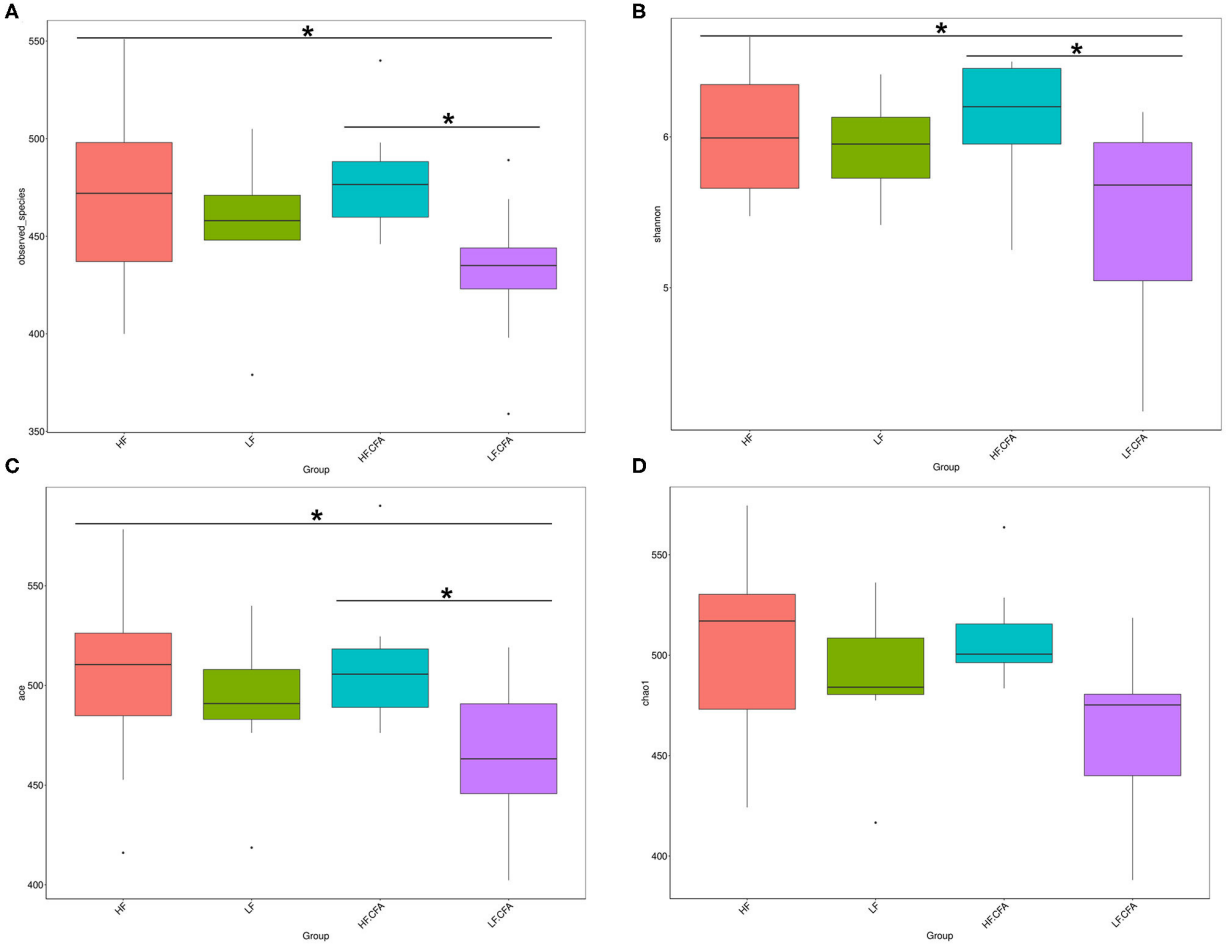
Microbiota alpha-diversity comparison among four groups, Piglets were regarded as the experimental units, each treatments with *n* = 9 except that HF.CFA with *n* = 8 due to a sample was eliminated. **(A–D)** Observed_species, Shannon index, ACE index, and Chao 1 index of four groups, respectively, HF, High fiber, no CFA; LF, Low fiber, no CFA; HF.CFA, High fiber with CFA challenge; LF.CFA, Low fiber with CFA challenge. *means *P* < 0.05.

**Figure 6:**
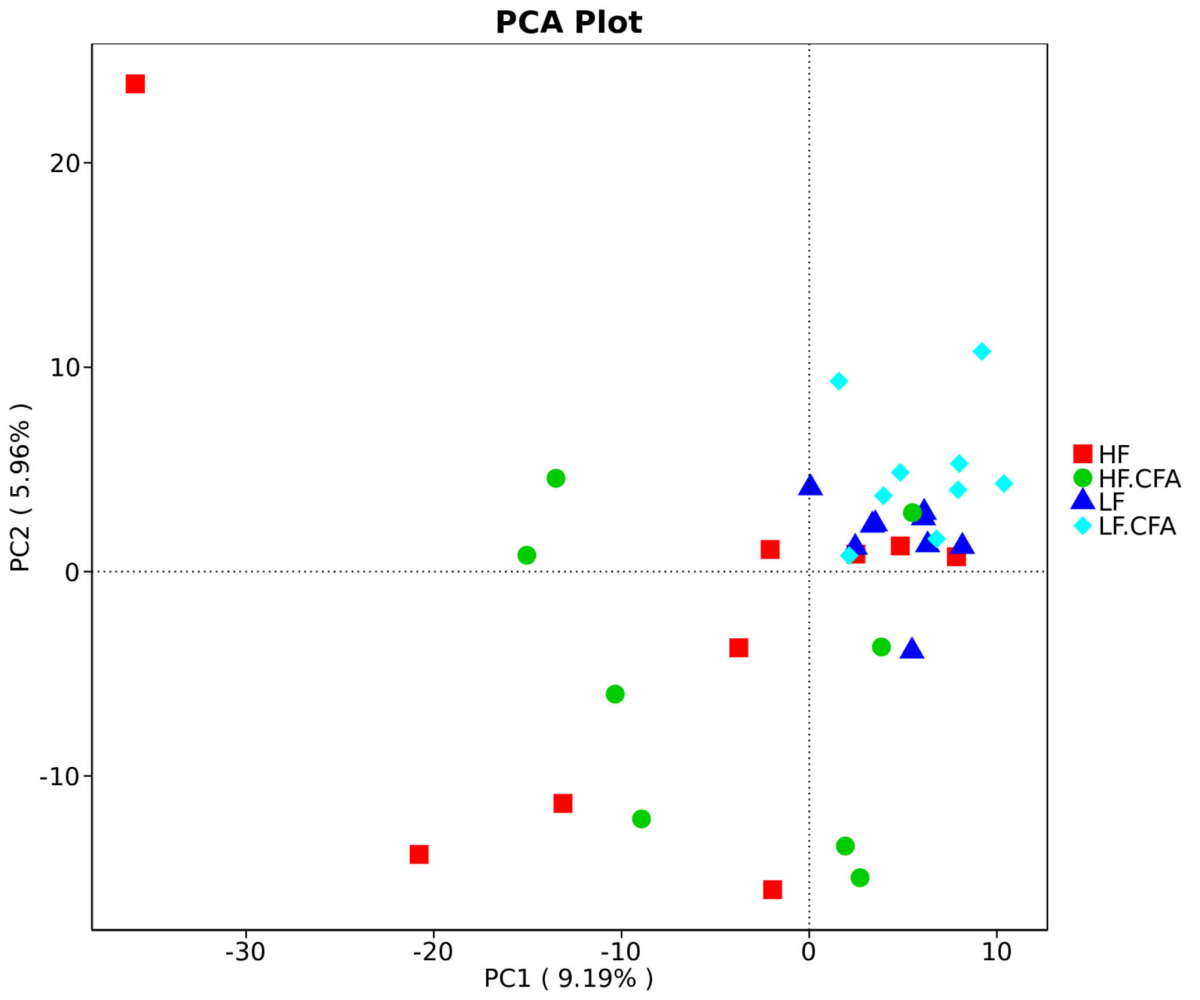
Comparison of the gut microbiota composition among four groups. Principal component analysis to visualize the euclidean distances of colon digesta samples from individual piglet. HF, High fiber, no CFA; LF, Low fiber, no CFA; HF.CFA, High fiber with CFA challenge; LF.CFA, Low fiber with CFA challenge.

To assess fecal microbial community structure, bacterial diversity (Shannon index), richness (observed species and Chao1 index), and ACE index for alpha diversity were investigated. Compared with piglets fed the LF diet, the observed species (Figure 5A, *p* = 0.01), Shannon index (Figure 5B, *p* = 0.03), and ACE index (Figure 5C, *p* = 0.02) increased significantly in piglets fed the HF diet, and there is a tendency of an increased Chao 1 index in piglets fed the HF diet (Figure 5D, *p* = 0.05). No interaction between dietary fiber and CFA treatment was observed on the above microbial parameters. Correspondingly, PCA was further carried out with dimensionality reduction analysis. The abundance of OUT showed that lack of dietary fiber resulted in obvious changes in the bacterial structure in the colon of piglets (Figure 6).

### Changes in Relative Abundance at the Phylum and Genus Levels and the Correlation Between Lung Pathology Scores and Phylum and Genera Levels

**Figure 7:**
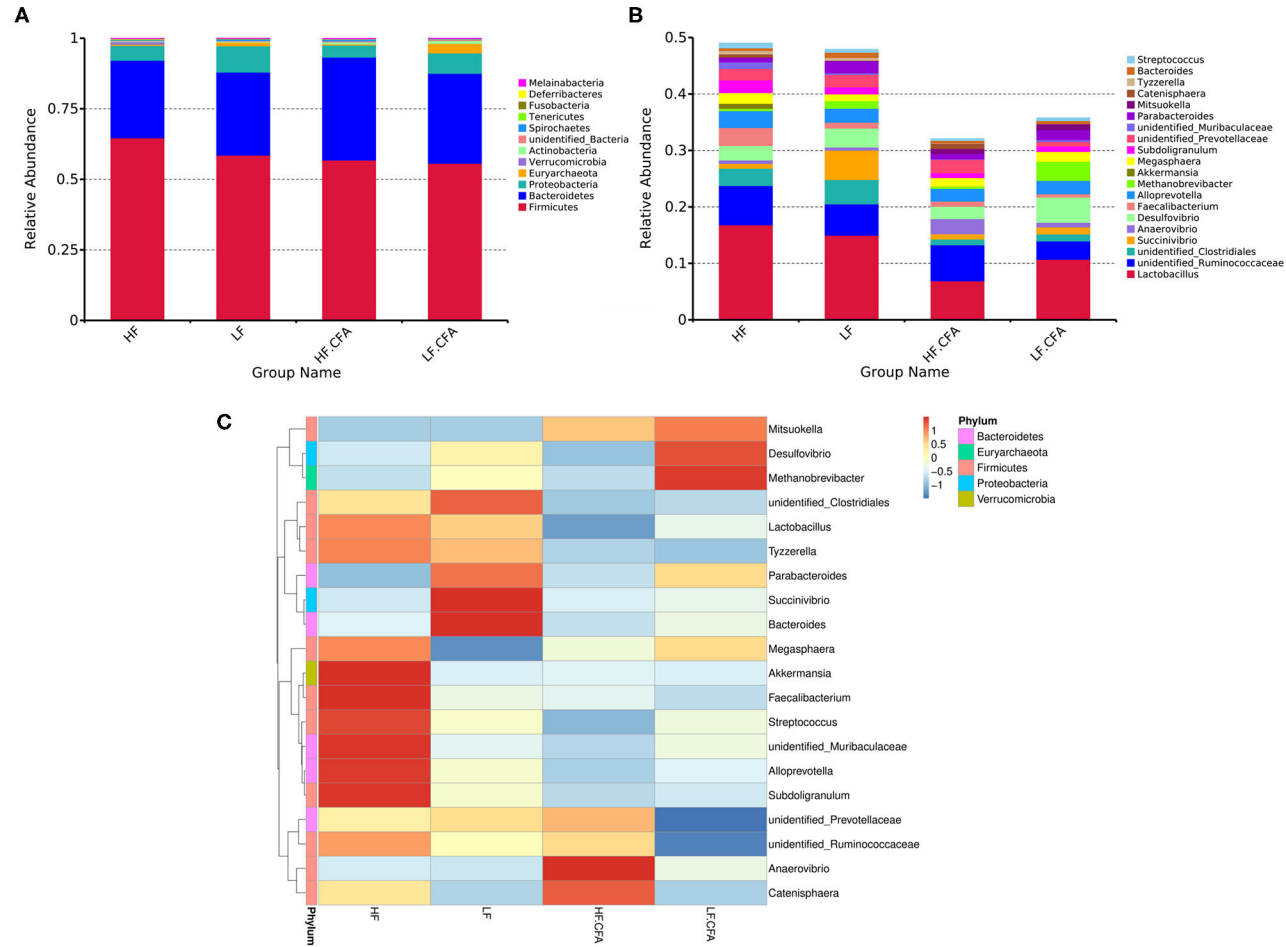
The histogram and heat map of the relative abundance of microorganisms at phylum level and genus level. **(A)** 16S rRNA gene analysis reveals that there are differences in the phylum level of three bacterial in the four groups. **(B)** 16S rRNA gene analysis reveals that there are differences in the genus level of six bacterial in the four groups. **(C)** Heat-map of species abundance based on top 20 genera clustering (vertical clustering). Different color means the different relative abundance of the genus in all the four treatments.

Effects of dietary fiber on the relative abundance of colonic microbiota at the phylum level were observed. The relative abundance of the top ten microbiotas is presented in Figure 7A. The top five dominated phyla were *Firmicutes, Bacteroidetes, Proteobacteria, Euryarchaeota*, and *Actinobacteria*. Feeding an HF diet decreased the relative abundance of *Euryarchaeota* (*p* < 0.01) and *Proteobacteria* (*p* = 0.03). The relative abundance of *Deferribactere*s was greater in piglets fed the HF diet (*P* = 0.02) than in piglets fed the LF diet.

The relative abundances at the genus level are presented in Figure 7B. Compared with LF diet, an HF diet increased the relative abundance of *unidentified_Ruminococcaceae* (*P* = 0.03) and *Catenisphaera* (*p* < 0.01) and tended to increase the relative abundance of *Faecalibacterium* (*p* = 0.08). An HF diet decreased the relative abundance of *Succinivibrio* (*p* < 0.01), *Desulfovibrio* (*p* = 0.02), and *Methanobrevibacter* (*p* < 0.01). It can be seen from the heat map that the relative abundances of *Akkermansia, Faecalibacterium, Streptococcus, unidentified_Muribaculaceae, Alloprevotella*, and *Subdoligranulum* were higher in HF diets without CFA, but after receiving CFA challenge, the relative abundance of *Anaerovibrio* and *Catenisphaera* increased significantly (Figure 7C). It is worth noting that the CFA challenge significantly increased the relative abundance of *Mitsuokella* (*p* = 0.03).

**Figure 8:**
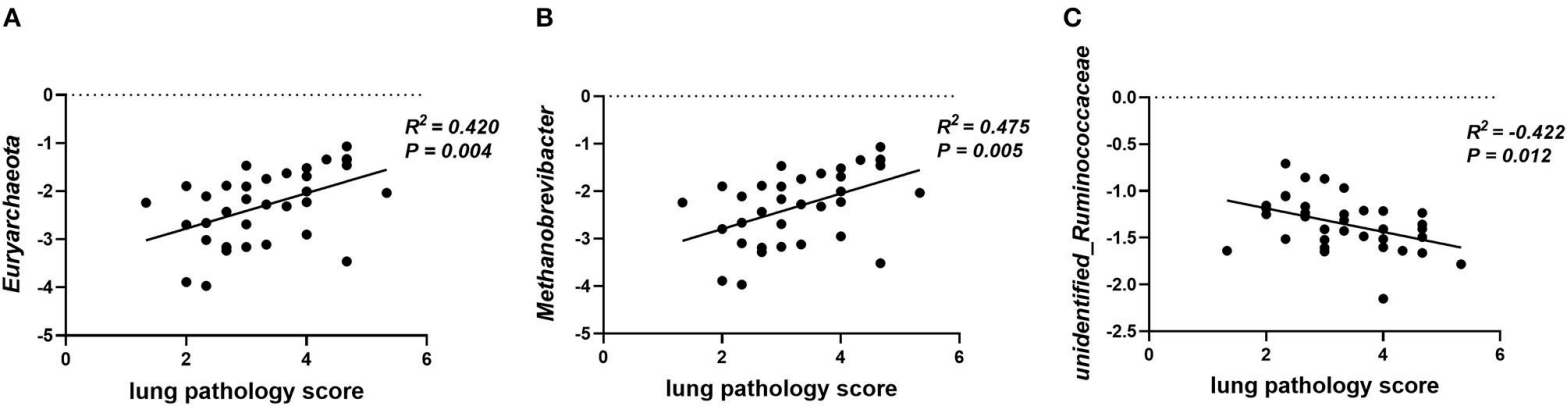
Plot of correlations between lung pathology score and gut microbial abundance at phylum and genus level. The correlation analysis was performed by Pearson correlation tests. **(A)** Lung pathology score is positively correlated with *Euryarchaeota* at the phylum level. **(B)** Lung pathology score is positively correlated with *Methanobrevibacter* at the genus level. **(C)** Lung pathology score is negatively correlated with *unidentified_Ruminococcaceae* at the genus level.

Gut microbiota participates in the regulation of the host's immunity. Thus, the correlation between lung pathology score and colonic microbial abundance at the phylum and genus level was investigated. The lung pathology score showed positive correlations with *Euryarchaeota* at the phylum level (Figure 8A, *p* = 0.04) and *Methanobrevibacter* at the genus level (Figure 8B, *p* < 0.01), but negative correlations with *unidentified_Ruminococcaceae* (Figure 8C, *p* = 0.01).

### Gene Expression in Lungs

**Figure 9:**
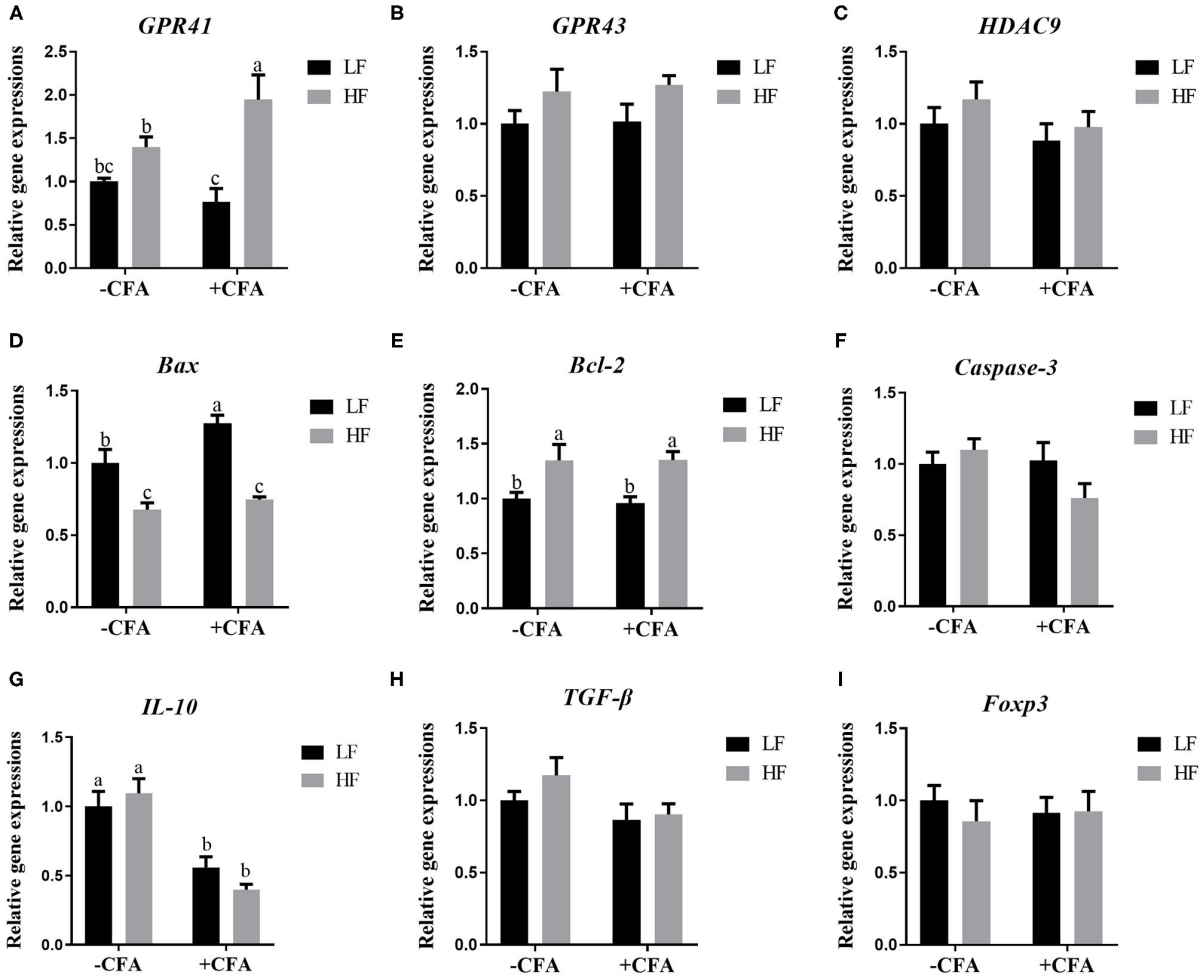
Relative mRNA abundance in the lung of piglets fed diets supplemented with fiber following CFA challenge. **(A)** GPR41, G protein-coupled receptor 41; **(B)** GPR43, G protein-coupled receptor 43; **(C)** HDAC9, Histone deacetylase 9; **(D)** Bax, BCL2-Associated X; **(E)** Bcl-2, B cell lymphoma 2; **(F)** Caspase-3, Cysteine-requiring aspartate protease 3; **(G)** IL 10, Interleukin 10; **(H)** TGF-β; transforming growth factor-β; **(I)** Foxp3, Forkhead box P3. Values are means, with their standard errors represented by vertical bars. ^a,b^Mean values with unlike letters were significantly different (*p* < 0.05).

**Figure 10:**
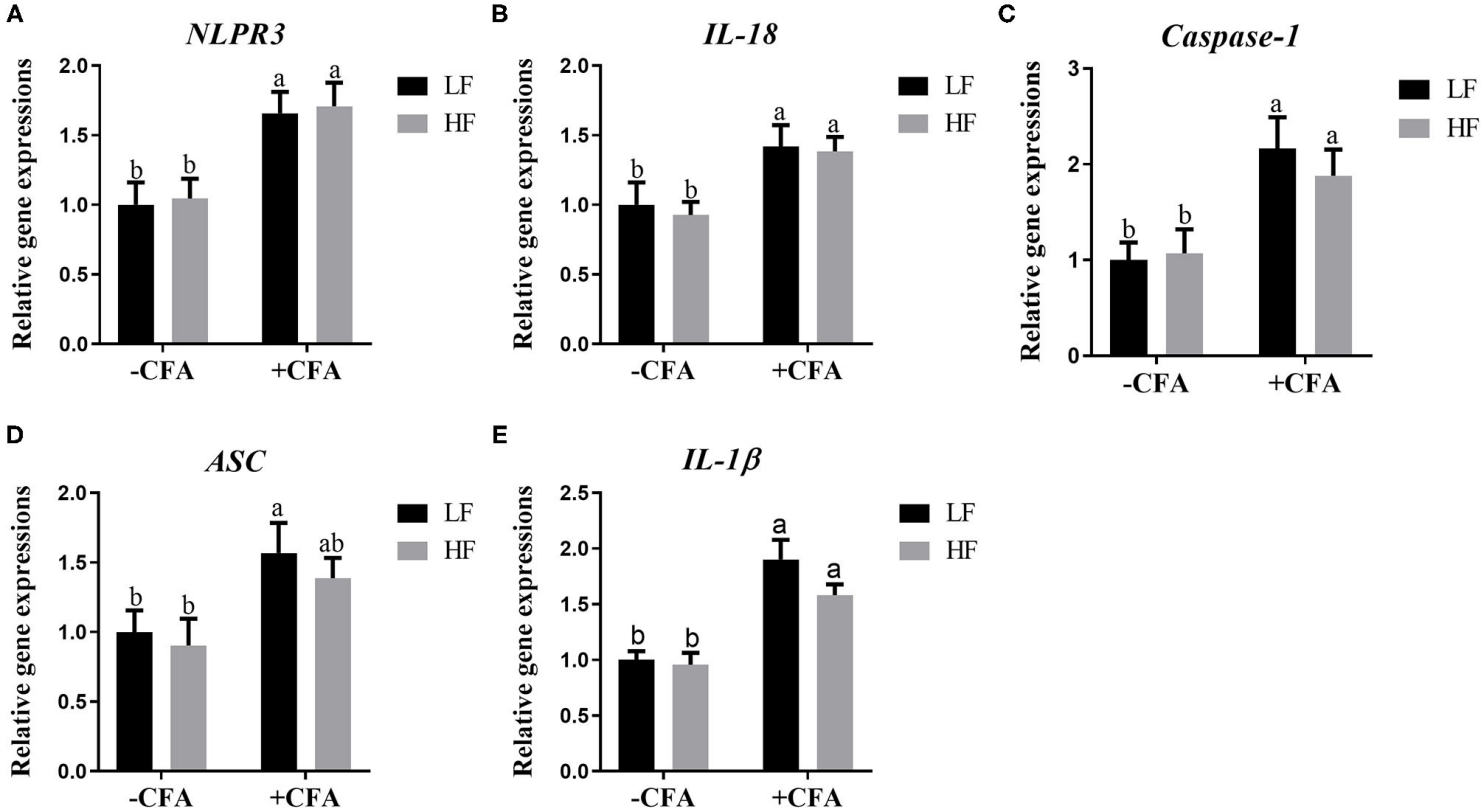
Relative mRNA abundance in the lung of piglets fed diets supplemented with fiber following CFA challenge. **(A)** NLRP3, Nod-like receptor; **(B)** IL-18, Interleukin 18; **(C)** Caspase-1, Cysteine-requiring aspartate protease 1; **(D)** ASC, Apoptosis-associated speck-like protein containing a CARD; **(E)** IL-1β, Interleukin 1β. Values are means, with their standard errors represented by vertical bars. ^a,b^Mean values with unlike letters were significantly different (*p* < 0.05).

The relative expression of the genes *GPR41* and *GPR43* in lung tissue samples were greater in piglets fed the HF diet than in the LF diet, and the relative expression of GPR41 was higher in piglets fed the HF diet and treated with CFA (Figure 9A, *p* < 0.05) than in those fed the LF diet and treated with CFA (Figures 9A,B, *p* < 0.05 and *p* = 0.07). Regardless of diet, the CFA challenge significantly increased the relative expressions of *Bax* and *Bcl-2* (Figures 9D,E, *p* < 0.05) and decreased the relative expressions of *IL-10* (Figure 9G, *p* < 0.05). The relative expression of *Bax* was lower in piglets fed the HF diet treated with CFA (Figure 9D, *p* < 0.05) than in piglets fed the LF diet and treated with CFA. The CFA challenge resulted in a greater expression of NLRP3 inflammasome-related genes (*NLRP3, IL18, Casp1, ASC*, and *IL1*β) in the lungs (Figures 10A–E, *p* < 0.05).

### Expression of Apoptotic Proteins in the Lung

**Figure 11:**
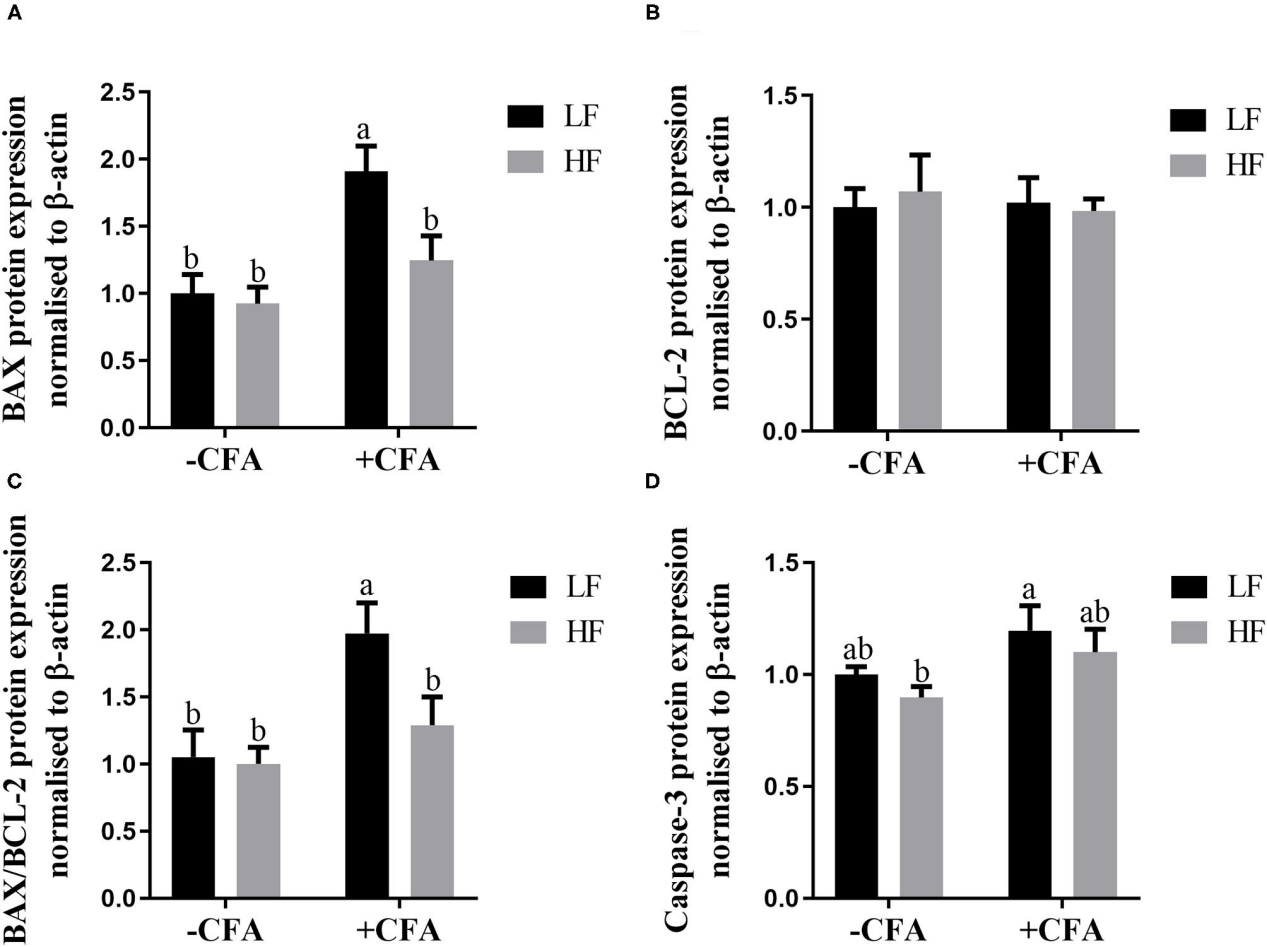
Relative protein expression of BAX **(A)**, BCL-2 **(B)**, BAX/BCL-2 **(C)**, and Caspase-3 **(D)** in lung of piglets fed diets supplemented fiber following CFA challenge. ^a,b^Mean value with unlike letters were significantly different (*p* < 0.05).

The expression of BAX (Figure 11A, *p* < 0.05) and BAX/BCL-2 (Figure 11C, *p* < 0.05) were lower in the lungs of piglets fed the HF diet and treated with CFA than in those piglets fed the LF diet and treated with CFA. Regardless of CFA treatment, piglets fed the HF diet had significantly higher expression of caspase-3 protein expressions in the lung (Figure 11D; Supplementary Figure S1, *p* < 0.05). There was no significant difference in the expression of BCL-2 protein across the treatments (Figure 11B).

## Discussion

Human respiratory health is closely related to the structure and function of the lungs. In developed and developing countries, far more people die of lung diseases than cancer and cardiovascular diseases (34). Many researchers have reported that alternation in intestinal microbes caused by imbalanced nutrition has been linked to increased risk of asthma and allergic diseases (35–37). Furthermore, SCFAs produced by the fermentation of dietary fiber by colonic symbiotic bacteria are known to have antiinflammatory effects (38–40). In the present study, we hypothesize whether SCFA produced by intestinal commensal bacteria can also play a positive role in the regulation of the lung immune stress of piglets. The results showed that piglets fed the LF diet had enhanced sensitivity of piglets to lung immune stress, stressing the important role of microbial metabolism of dietary fiber on the protection of lung function under the immune challenge.

This experiment established a pulmonary immune stress model by CFA. Previous studies have revealed that the non-infectious pulmonary immune stress model of piglets was successfully induced by intravenous injection of CFA (41, 42). As a non-specific inflammatory marker, C-reactive protein is directly involved in the body's inflammatory response (43). In this study, respiratory rate and serum C-reactive protein were significantly elevated in piglets challenged by CFA, indicating that immune stress was present in the lungs. After the CFA challenge, the interstitial hyperplasia of piglets widens, the number of alveoli decreases sharply, the alveolar cavity is severely narrowed, and there is a large amount of inflammatory cell infiltration. The NOD-like receptor NLRP3 is an intracellular pattern recognition receptor that can be activated by a variety of different exogenous and endogenous stimuli to assemble a multimolecular protein complex, the “NLRP3 inflammatory body” (44). NLRP3 is a key component of the inflammasome complex. Upon priming of the cells, the NLRP3 inflammasome complex is formed with the help of the adaptor protein ASC that promotes NLRP3-mediated cleavage and activation of caspase-1. Caspase-1 is the central stimulator promoting the maturation of pro-IL-1β and pro-IL-18, thereby promoting the shear maturation and secretion of proinflammatory cytokines IL-1β and IL-18, causing a series of inflammatory reactions (45, 46). The inflammasome can sense the danger/stress signal, leading to increased levels of active IL-1β (47). Furthermore, the role of IL-1β as a critical inflammatory mediator of acute inflammation and tissue remodeling has been well-established (48). Studies have found that mice have elevated levels of caspase-1 in lung tissue after being stimulated by a cigarette smoker (49). The level of caspase-1 in lung tissue of patients with COPD and smokers is also higher than that of the non-smokers (50). Endogenous IL-18 plays a role in the inflammatory response by causing an increase in the production of cytokines and chemokines. Studies have found that *in vitro* and exogenous administration of IL-18 increased neutrophil recruitment and also vascular permeability, which is a mark of lung injury. Conversely, IL-18 blockade by neutralizing Ab reduced the severity of lung inflammation (51). In this experiment, the relative mRNA expression of *NLRP3, ASC, Caspase-1, IL-1*β, and *IL-18* in the lungs of piglets increased after the CFA challenge, further indicating that immune stress has occurred in the lungs.

Metabolism of dietary fiber occurs in the gastrointestinal tract and requires intestinal flora. Therefore, the metabolites released by the gut flora represent a key signal for maintaining host–microbe symbiosis. However, the academic community currently lacks data clarifying the specific mechanisms involved in the intestine–pulmonary axis. We aimed to establish an association between the intestinal microflora and lung immunity and hypothesized that this association may mediate the effect of microbial metabolism of dietary fiber on lung health. We found that the effect of dietary fiber on pulmonary immune stress may be related to the production of SCFAs.

There is increasing evidence that a diet rich in fiber helps maintain a healthy gut microbiota and increases microbial diversity and function. With the industrialization of diets, low fiber intake has led to a decline not only in the intestinal bacterial diversity but also in functional changes, including a decline in SCFA production, and the emergence of chronic inflammatory diseases. A previous study observed that high fiber intake stimulated intestinal bacteria to produce SCFAs, enhanced the production of mucous and antibacterial peptides, and increased the expression of tight junction proteins. SCFAs could reduce the oxygen content to maintain immune function (52). SCFAs are found in high concentrations in the caecum and proximal colon and are used as energy in colon cells (especially butyrate) but can also be transported through the portal vein to the peripheral circulation, where they act on the liver and peripheral tissues (53). Although levels of SCFAs in peripheral blood are low, they are widely involved in the body's immune regulation, glucose and lipid metabolism process, and satiety regulation (54). In the present study, we found that microbial alpha diversity (observed_species, Shannon, Chao1, ACE indices) increased in piglets fed the HF diet. The higher diversity of gut microbiota was associated with greater plasticity in response to immunity (55) and has been used as a health indicator (56). Thus, increased microbial diversity induced by dietary fiber may exert a positive effect on health. Moreover, the PCA chart showed significant clustering of samples dependent on diet, which further indicates that dietary fiber has a profound effect on gut microbes. In addition, we found that an HF diet significantly increased the thickness of the colonic mucosa of piglets. It has been demonstrated that in the absence of dietary fiber, the intestinal flora uses host mucus as a source of nutrients, leading to erosion of the colonic mucous barrier, reducing the thickness of the colonic mucosa, and further increasing the risk of infection (57). Thus, the enhanced α-diversity and colonic mucous thickness might lead to greater plasticity and favorable environment for lung immune stress in piglets fed the HF diet.

Compared to piglets fed the LF diet, a lower relative abundance of *Proteobacteria* and *Euryarchaeota* and a higher relative abundance of *Deferribacteres* were found in the colonic contents of piglets fed the HF diet. The increase in *Proteobacteria* is a frequent phenotype in inflammatory disease-associated dysbiosis (58). Patients with COPD, whose intestinal and airway microbiota are dysregulated, often exhibit more abundant overrepresentation of *Proteobacteria* (59). In addition, *Proteobacteria* are associated with intestinal inflammation (60), and high proportions of bacteria belonging to this phylum are known to result in gut pathology (61). The improved colon morphology and lower abundance of *Proteobacteria* observed in the colonic contents of piglets fed the HF diet suggested that these bacteria support the development of the colonic function of piglets. In addition, a recent study found that *Deferribacteres* protected mice against enteric Salmonella infection by interfering with pathogen invasion and virulence factor expression (58). We also analyzed the relative abundance of microbes at the genus level and found that higher dietary fiber intake increased the relative abundance of *unidentified_Ruminococcaceae* and *Catenisphaera*, and decreased the relative abundance of *Succinivibrio, Desulfovibrio*, and *Methanobrevibacter*. *Methanobrevibacter* is a methanogenic bacterium, which falls within the phylum *Euryarchaeota*. The added fiber led to a decrease in the abundance of methanogenic flora and methane production and reduced the occurrence of colon cancer (62). Moreover, these two bacteria are also positively correlated with lung pathology scores in the present study. SCFA-producing *Ruminococcacea* families play a role in maintaining intestinal immune homeostasis (63, 64), and studies have reported the importance of those in the amelioration of chronic inflammatory diseases and the promotion of colonocyte health (65, 66). In addition, the relative abundance of *unidentified_Ruminococcaceae* is also negatively correlated with lung pathology score. Therefore, an HF diet may reduce the relative abundance of *Euryarchaeota* and *Methanobrevibacter* and increase the relative abundance of *unidentified_Ruminococcaceae* to alleviate lung immune stress. The abundance of *Desulfovibrio* is significantly increased in steatosis, steatohepatitis, and chronic kidney disease (67, 68), which indicated that *Desulfovibrio* exacerbates the occurrence of inflammation.

SCFAs are the main metabolites formed by the fermentation of dietary fiber in the hindgut. In the present study, an LF diet decreased the concentration of acetate and propionate in the contents of the colon, which are consistent with the previous research (69). fiber intake and SCFAs affect not only the intestinal health but also the physiological functions of the lungs (70). For example, grain fiber intake is inversely proportional to the risk of COPD (71). A prospective study found that long-term low fiber feeding can aggravate allergic airway disease (AAD) in mice but can be corrected by supplementing drinking water with propionate (25). Similarly, another study found that a high fiber diet altered the composition of the microbiota, increased the production of SCFAs (specifically acetate), and prevented AAD development in adult mice. Results are now available that changes in the gut microbiome are related to the pathogenesis of various diseases. Attributing to the close relationship between intestinal flora composition and diet regimen (72, 73), insufficient dietary fiber intake may increase susceptibility to inflammatory diseases (74).

The SCFAs can participate in the immune response through signal transduction through G protein-coupled receptors (GPCRs). The main GPCRs activated by SCFAs are GPR41 and GPR43. GPR43 is highly expressed in mouse hematopoietic tissues such as bone marrow and spleen cells, indicating that GPR43 plays a potential role in regulating the development or differentiation of immune cells (75). The activation of GPR43 by acetate has been shown to be involved in the prevention of colitis and arthritis (38). Like GPR43, GPR41 also recognizes SCFAs, but to a lesser extent (76). In the present study, the piglets fed the HF diet had significantly increased expression of the gene encoding GPR41 in the lungs, and GPR43 also had a tendency to be affected by the HF diet. These two receptors can be expressed in different cell subgroups or tissues to affect different aspects of the immune response. Dietary fiber influences the overall magnitude of the inflammatory response against HDM (dust mite extract) and found that the beneficial effects of propionate pretreatment on the HDM challenge induced eosinophilic infiltration in lungs were lost in GPR41-deficient mice, indicating that GPR41 is essential for the protective effect of propionate (25).

The Bcl-2 family proteins play an important role in regulating the mitochondrial apoptosis pathway (77). Caspase-3 is one of the most important members of the cysteine protease family and is the key effector of apoptosis and the final determinant of apoptotic death. Previously, it was found that the relative expression of *Bax* mRNA in lung tissue was upregulated in response to diffuse alveolar injury, and that acetate, propionate, and butyrate stimulated the expression of BCL-2 protein but inhibited the expression of Bax protein expression (78). In the present study, the relative expression of *Bax* mRNA, BAX protein, and caspase-3 protein was increased in the lung tissue of piglets fed the LF diet treated with CFA. However, the relative expression of *Bax* mRNA decreased, and BAX and caspase-3 protein levels were not altered in piglets fed the HF diet, suggesting that the HF diet reduced the degree of apoptosis caused by immune stress in the lungs. Of note, the occurrence of apoptosis is highly correlated with the inflammation process (79–81). The decreased apoptosis may indicate the attenuated immune stress response.

The benefits of dietary fiber on the attenuation of CFA-induced lung stress could be attributed to alternations of immunity because we observed that dietary fiber significantly reduced the eosinophilic infiltration in the lungs. Our original hypothesis was that dietary fiber can stimulate Treg cell signaling pathways to alleviate lung immune stress by increasing IL-10 and TGF-β. We have detected the mRNA expressions of IL-10 and TGF-β in lung tissues. No significant effect of dietary fiber on these cytokines was observed, suggesting that dietary fiber appeared to attenuate the lung immune stress in a Treg-cell independent way. However, this conclusion should be drawn with caution, since the population and function of Treg cells in the lungs were not clarified due to the limitations of collagenase and antibodies for pigs; thus we cannot confirm this issue at present. The mechanism mediating the gut microbial metabolism of dietary fiber on immunity is quite complicated, and dietary fiber may exert its effect through non-Treg cell pathways. A recent study showed that high dietary fiber can protect mice from influenza virus infection by preventing immune-mediated pathology and antiviral T-cell responses (82). Other reports also found the involvement of SCFAs in regulating immune cell metabolism by the conversion of SCFAs into acetyl-CoA and integration into the Krebs cycle (83–85). SCFAs can also influence T-helper cell differentiation *via* mTOR signaling (85) or accelerate recall responses of memory CD8+ T cells (86). Considering the complexity of the microbiota and their metabolites SCFAs on immunity, further studies are needed to clarify the exact mechanism(s) mediating the effects of dietary fiber on the lung immunity in a pig model.

Collectively, our results showed that dietary fiber supplementation improved the outcome of lung immune stress, which may be associated with microbiota and their production of SCFAs.

## Data Availability Statement

The datasets presented in this study can be found in online repositories. The names of the repository/repositories and accession number(s) can be found in the article/Supplementary Material.

## Ethics Statement

The animal study was reviewed and approved by the Animal Care and Use Committee of the Animal Nutrition Institute of Sichuan Agricultural University (No. SICAU-2015-034).

## Author Contributions

DW, YY, and YZ conceived the project and designed the experiments. YY, XJ, XC, LZ, WL, BF, YL, SX, ZF, JL, and XZ performed the experiments. YY and YZ analyzed the data and wrote the paper. All authors have read and approved the final manuscript.

## Funding

This work was supported by the Projects of the National Natural Science Foundation of China (Grant number 32102589).

## Conflict of Interest

The authors declare that the research was conducted in the absence of any commercial or financial relationships that could be construed as a potential conflict of interest.

## Publisher's Note

All claims expressed in this article are solely those of the authors and do not necessarily represent those of their affiliated organizations, or those of the publisher, the editors and the reviewers. Any product that may be evaluated in this article, or claim that may be made by its manufacturer, is not guaranteed or endorsed by the publisher.
