# Supplementary material for: Deprivation of Dietary Fiber Enhances Susceptibility of Piglets to Lung Immune Stress
Source: Front Nutr. 2022 Feb 10;9:827509. doi: 10.3389/fnut.2022.827509 (PMC8867169; doi:10.3389/fnut.2022.827509)
Supplement: Supplementary file 1 [file Data_Sheet_1.docx]

**Supplemental data**

Table S1

Effect of fiber supplementation on growth performance of piglets^1,2,3,4^

| Item | Diet | | SEM | P-value |
| --- | --- | --- | --- | --- |
|  | LF | HF |  |  |
| No. of piglet | 18 | 18 | - | - |
| Body weight, kg |  |  |  |  |
| d 0 | 9.68 | 9.75 | 0.260 | 0.840 |
| d 7 | 11.49 | 11.73 | 0.352 | 0.634 |
| d 14 | 14.02 | 14.56 | 0.453 | 0.400 |
| d 21 | 17.14 | 18.17 | 0.692 | 0.302 |
| d 28 | 20.60 | 21.65 | 0.822 | 0.374 |
| Average daily feed intake, kg/d |  |  |  |  |
| d 1 - 7 | 0.39 | 0.45 | 0.028 | 0.096 |
| d 7- 14 | 0.57 | 0.67 | 0.043 | 0.115 |
| d 14- 21 | 0.72 | 0.85 | 0.062 | 0.149 |
| d 21- 28 | 0.81 | 0.89 | 0.043 | 0.209 |
| d 1- 28 | 0.63 | 0.72 | 0.039 | 0.100 |
| DE intake, Mcal |  |  |  |  |
| d 1 - 7 | 1.52 | 1.59 | 0.101 | 0.624 |
| d 7- 14 | 2.20 | 2.30 | 0.155 | 0.641 |
| d 14- 21 | 2.78 | 2.94 | 0.226 | 0.622 |
| d 21- 28 | 3.14 | 3.08 | 0.157 | 0.791 |
| d 1- 28 | 2.41 | 2.48 | 0.141 | 0.732 |
| Lys intake, % |  |  |  |  |
| d 1 - 7 | 0.55 | 0.55 | 0.036 | 0.957 |
| d 7- 14 | 0.80 | 0.80 | 0.055 | 0.961 |
| d 14- 21 | 1.01 | 1.02 | 0.080 | 0.907 |
| d 21- 28 | 1.14 | 1.07 | 0.056 | 0.397 |
| d 1- 28 | 0.87 | 0.86 | 0.050 | 0.876 |
| Ca intake, % |  |  |  |  |
| d 1 - 7 | 0.28 | 0.30 | 0.019 | 0.531 |
| d 7- 14 | 0.40 | 0.43 | 0.029 | 0.578 |
| d 14- 21 | 0.51 | 0.54 | 0.042 | 0.564 |
| d 21- 28 | 0.58 | 0.57 | 0.029 | 0.871 |
| d 1- 28 | 0.44 | 0.46 | 0.026 | 0.643 |
| P intake, % |  |  |  |  |
| d 1 - 7 | 0.18 | 0.18 | 0.011 | 0.600 |
| d 7- 14 | 0.26 | 0.27 | 0.026 | 0.700 |
| d 14- 21 | 0.32 | 0.34 | 0.018 | 0.657 |
| d 21- 28 | 0.37 | 0.36 | 0.017 | 0.720 |
| d 1- 28 | 0.28 | 0.29 | 0.017 | 0.833 |

^1^ Values are means with pooled SEM, n=18 in each group

^2^ before being challenged, it was divided into two treatment groups (LF group and HF group)

^3^ ADFI average daily feed intake

^4^ DE means digestible energy; Ca means calcium; P means phosphorus; LF means low dietary fiber; HF means high dietary fiber

^5^ Different letter ^a, b^ denotes *P <* 0.05

Table S2

Effect of dietary fiber on morphology and colonic mucosal thickness of small intestine in piglets

|  | Diet | | SEM | *P*-value |
| --- | --- | --- | --- | --- |
| Item | LF | HF |  |  |
| Duodenum |  |  |  |  |
| Villus height (μm) | 488.92 | 476.00 | 23.759 | 0.705 |
| Crypt depth (μm) | 228.70 | 236.10 | 12.004 | 0.667 |
| VCR | 2.16 | 2.07 | 0.073 | 0.402 |
| Jejunum |  |  |  |  |
| Villus height (μm) | 434.49 | 474.36 | 23.526 | 0.239 |
| Crypt depth (μm) | 194.77 | 192.77 | 9.530 | 0.883 |
| VCR | 2.26 | 2.55 | 0.112 | 0.086 |
| Ileum |  |  |  |  |
| Villus height (μm) | 328.39 | 370.27 | 14.149 | 0.044 |
| Crypt depth (μm) | 171.08 | 162.81 | 5.190 | 0.267 |
| VCR | 1.93 | 2.33 | 0.102 | 0.013 |

Mean values with their standard error, n = 9 in each group

VCR = villous height to crypt depth ratio.

Fig. S1


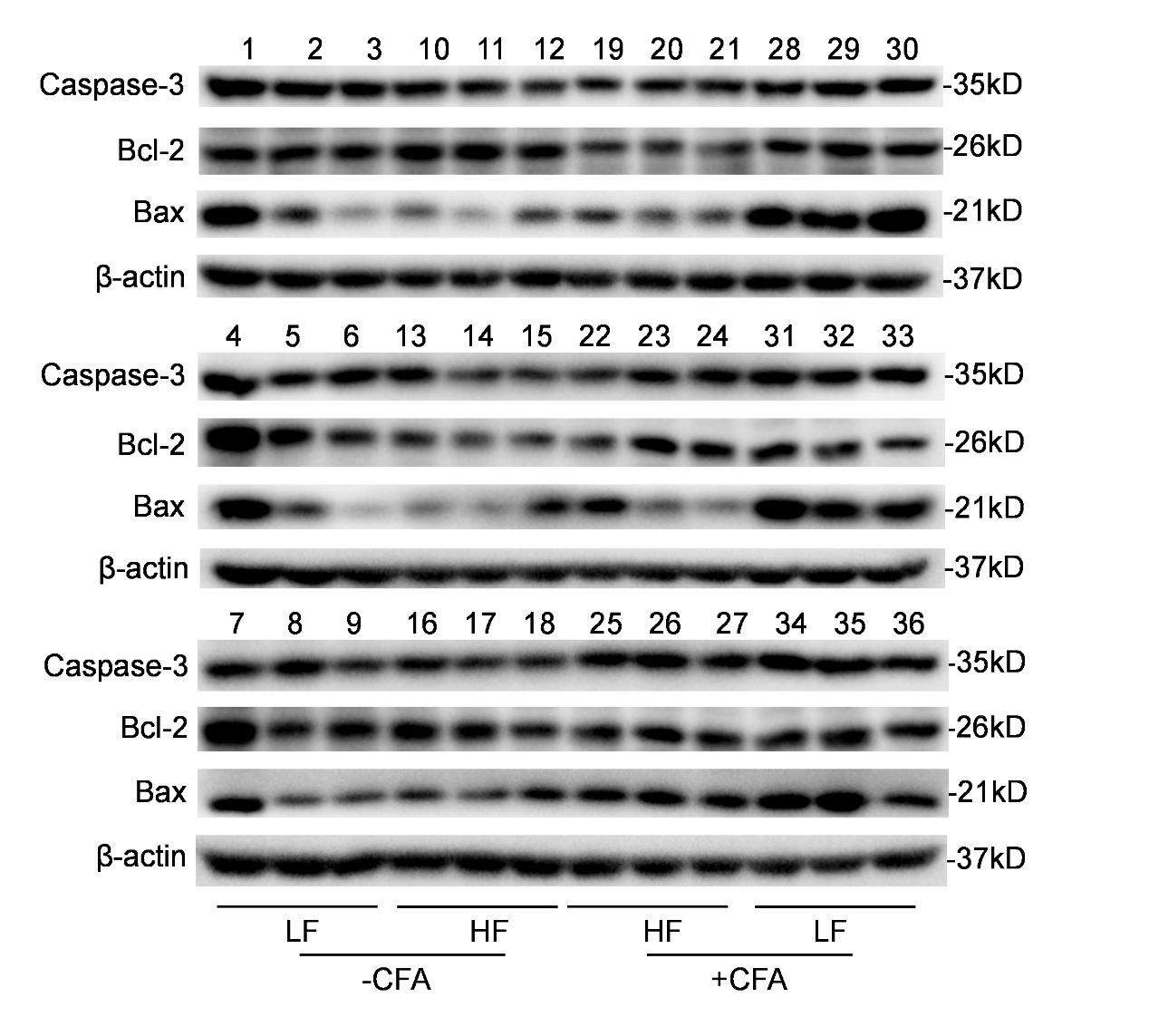


Fig. S1. Protein blots of BAX, BCL-2 and Caspase-3 in western blot studies. Each group tested nine samples in three batches(n=9).
